# IL35 Production After Living-donor Renal Transplantation: Relevance to Local, Exosome-mediated, Clinical Tolerance

**DOI:** 10.1097/TXD.0000000000001980

**Published:** 2026-07-06

**Authors:** William J. Burlingham, John H. Fechner, Ewa Jankowska-Gan, Sudipta Tripathi, Brittany L. Schreiber, Anil Chandraker, Dixon B. Kaufman, David P. Foley

**Affiliations:** 1 Division of Transplantation, Department of Surgery, School of Medicine and Public Health, University of Wisconsin-Madison, Madison, WI.; 2 Renal Division, Brigham and Women’s Hospital, Harvard Medical School, Boston, MA.

## Abstract

**Background.:**

We previously reported that 100% of HLA-identical siblings and 50% of HLA-haploidentical donor-recipient pairs exhibited bidirectional immunoregulation (BDIR) before living-related renal transplantation. BDIR predicted successful transplant outcomes. However, at the time, we were unaware of the role of the cytokine interleukin-35 (IL35) in immunoregulation, and we now appreciate exosomes as a means of confining immunoregulation to the transplanted tissue. Here we show that HLA-identical sibling transplants use IL35 in posttransplant BDIR.

**Methods.:**

We analyzed the posttransplant IL35 response using the trans-vivo delayed type hypersensitivity assay in HLA-identical sibling transplants, using anti-IL35 antibodies to block immune-regulatory function as measured by either (1) inhibition of the tetanus toxoid/diphtheria toxoid recall response or (2) uncovering of direct response, in both the donor anti-recipient and the recipient anti-donor (minor H) antigen directions.

**Results.:**

We found that minor antigen-specific human regulatory T cells, known to be present in peripheral blood mononuclear cell of HLA-identical siblings pretransplant, also caused BDIR via production of IL35 posttransplant.

**Conclusions.:**

We conclude that human IL35 is a component of BDIR in HLA-identical sibling transplantation. Since IL35 is one of a subclass of exosome-delivered cytokines, this means that human IL35 could sustain local BDIR within the transplant itself. It follows that local BDIR would persist indefinitely if donor leukocytes were allowed to stably engraft. This may account for the success of combined total lymphoid irradiation/bone marrow transplantation/renal transplantation induction of stable tolerance in HLA-identical sibling transplant recipients.

## INTRODUCTION

Both immunogenic and immunoregulatory products of allografts have now been defined as components of small extracellular vesicles (sEVs), that is, exosomes.^1,2^ On the immunogenic side, the production of graft-specific alloantibodies was recently found to arise not from passenger cell migration to host lymph nodes, but rather from export of sEV from the transplant to subcapsular sinus macrophages that transport them to alloreactive B cells, with a critical role for sEV-associated complement and IgM.^3,4^ On the immunoregulatory side, a variety of sEV-transported products of regulatory T cells have been recently described, including interleukin-35 (IL35),^1^ the ectoenzymes CD39 and CD73, which convert extracellular ATP to adenosine,^5^ and more recently, transforming growth factor beta-1 (TGFβ1).^6^ Interestingly, none of these exosomal products are immediately immunosuppressive; rather, as exosome components, they are initially inactive. They become immune-suppressive only when they either convert their substrate (extracellular ATP) to an immunosuppressive derivative (adenosine) in the case of CD39/CD73 or when latent cytokines are activated on the surface of the exosome-acquiring cell. This can occur because of either (1) conversion to an active 1:1 subunit form from the inactive 2:1 subunit form when tetraspanin CD81-bound IL35 is transported to the cell surface^1^ or (2) release from a membrane-bound latency complex consisting of TGFβ1, latency-associated peptide, and glycoprotein A repetitions predominant, associated mainly with CD81 exosomes, in the case of TGFβ1.^6^ Importantly, CD39/73, IL35, and TGFβ1 all require an activation process.

In a previous publication, we identified a new parameter that predicted kidney transplant success in living-related donor-recipient (D-R) pairs: bidirectional immunoregulation (BDIR).^7^ This was defined as the presence, before transplant, of 2 regulated responses: (1) by the recipient toward the donor alloantigens and 2) by the donor toward the recipient alloantigens. These bidirectional regulations were primarily because of prior exposure to noninherited maternal antigens^8^ in the case of sibling-to-sibling transplants.

Our purpose here was to determine the contribution of IL35 to posttransplant bidirectional regulation. We propose that when HLA-identical (HLA-ID) kidney transplantation is amplified by donor hematopoietic stem cell (HSC) co-transplantation, donor T cells perpetuate local “infectious” tolerance, allowing for indefinite graft survival.

## MATERIALS AND METHODS

### Study Population

Immunologic monitoring was performed on blood samples obtained from healthy kidney donors (all males) and their HLA-ID sisters (recipients). Patients and donors were recruited according to informed consent procedures, subject to Institutional Review Board approval at the University of Wisconsin-Madison in Madison, WI. Peripheral blood was obtained 2–10 y after transplantation for end-stage renal disease. All bloods were processed by Ficoll-Hypaque to enrich for white blood cells. A portion of each leukocyte sample was sonicated for antigen preparation, while the remaining cells were used as responders in the trans-vivo delayed type hypersensitivity (tv-DTH) assay.

### tv-DTH Analysis

Reciprocal analysis of recipient anti-donor and donor anti-recipient immune responses was done using the tv-DTH technique with CB17.SCID mice footpad injection and readout by caliper at 24 h as described previously.^7^ This technique is especially useful for measuring immune responses to minor H antigens.^9^ Both tetanus toxoid (TT) and diphtheria toxoid (DT) were used for a positive control. A combination of 10 mg of mouse anti-human IL-12 p35 subunit (p35; clone 27537 R&D Systems), plus 10 mg of a mouse anti-mouse Epstein-Barr virus-induced gene 3 (Ebi3) monoclonal antibody (weakly cross-reactive with human Ebi3; Shenandoah Biotechnology) was co-injected along with human peripheral blood mononuclear cell plus antigen (10 μL of a sonicate of 5 × 10^6^ cells). To measure the inhibition response, antigen plus TT/DT were co-injected and compared with positive control anti-TT/DT recall response alone. A similar combination of anti-IL35 antibodies was used to reverse immunosuppression. Data were analyzed using a paired Student *t* test.

## RESULTS

**FIGURE 1.**
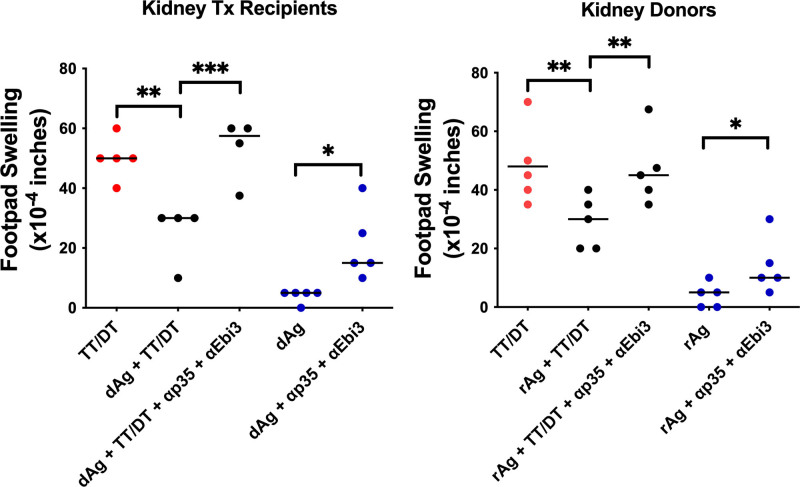
Bidirectional immunoregulation between HLA-ID recipient lymphoid cells is IL35-dependent. Recipient anti-HLA-ID donor tv-DTH response (left); donor anti-HLA-ID recipient tv-DTH response (right). In both cases, the response to mHAg mismatches of the sibling caused significant suppression of the recall response to TT/DT (donor, left; recipient, right). The recall response to TT/DT was completely restored by the inclusion of anti-IL35 antibodies along with the mHAg-mismatched donor (left) or recipient (right) antigen preparation. Paired 1-way Student *t* test used for all comparisons except for the recipient response to dAg vs dAg + αp35 + αEbi3. *P < 0.05;**P < 0.01;***P < 0.001. αEbi3, anti-Ebi3; αp35, anti-p35; dAg, donor antigen; DT, diphtheria toxoid; HLA-ID, HLA-identical; IL-35, interleukin-35; mHAg, minor histocompatibility antigen; rAg, recipient antigen; TT, tetanus toxoid; tv-DTH, trans-vivo delayed type hypersensitivity; Tx, transplant; αEbi3, anti-Ebi3; αp35, anti-p35.

We analyzed kidney transplant patients with the highest degree of HLA matching, namely HLA-ID siblings, known to benefit uniformly from pretransplant BDIR.^7^ As shown in Figure 1, we found that sisters and their kidney transplant HLA-ID (brother) donors continue to exhibit BDIR posttransplant, as measured by both inhibition of their TT-specific tv-DTH response (*P* < 0.01) and by the uncovering of a direct response to their sibling (*P* < 0.01). In both measurements of posttransplant BDIR, blocking IL35 subunits could reverse suppression (*P* < 0.01).

## DISCUSSION

Here, we provide evidence that in humans, IL35 is part of host and donor mutual regulation after HLA-ID kidney transplants. Our previously published analysis of mouse IL35 showed that it was produced in an inactive, precursor form that was associated with exosomes via the tetraspanin CD81,^1^ and that it promoted infectious tolerance by stimulating nonregulatory T cells to produce IL35 and promoting B- and T-cell exhaustion.^1^

We propose that human IL35, like mouse IL35, is secreted as an exosome-associated precursor, along with H_2_O-soluble subunits Ebi3 and IL12α, which themselves are immunosuppressive.^10^ CD81-associated “pre-IL35” was suggested by Burlingham and Chandraker (unpublished) as well as others (M. Feige, personal communication). Human “mixed” cell lines produced CD73^−^mediated adenosine-based immunosuppression^11^ that Schneider et al ^12^ attributed to exosomes that could be elaborated by human CD8 T cells. In mice, exosomes carrying a CD81^−^associated form of the cytokine resulted in membrane-bound active IL35 causing immunosuppression and upregulating programmed cell death protein 1/lymphocyte activation gene-3 checkpoint proteins.^13^ Interestingly, uptake of IL35 was also found to occur on bystander T cells (CD4>>CD8) and B cells located in areas of exosomal pre-IL35 release.^1^ Other immune cell-derived immunosuppressive cytokines have now been added to the exosomal-derived (hence “local”) category in mouse^5,6,12,14^ and human^15^ studies.

Can the strategy of exosome-mediated immunosuppression be applied to allografts, so as to provide local immune protection of organ transplants? Indeed, local suppression of immune response to the transplanted organ has been suggested as a possible long-term solution to health problems caused by the continuous systemic administration of immunosuppressive drugs over a lifetime.

**FIGURE 2.**
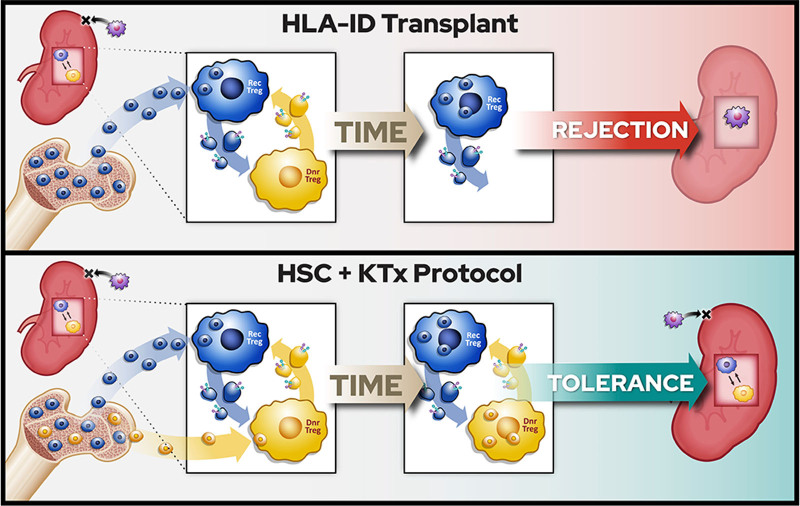
Long-term loss of bidirectional immune regulation in HLA-ID KTx and rescue by simultaneous bone marrow + KTx (top). Initially, recipient (blue) plus “residual” donor Treg cells (yellow) in the kidney produce inhibitory exosomes and prevent Teff cell access; but ultimately, bi-D regulation subsides because of a lack of replacement of donor T cells, allowing access of recipient Teff cells to the graft and rejection. Combining donor HSC with KTx under TLI coverage results in a sustained source of bone marrow-derived donor Treg cells, sustaining intragraft bi-D immunoregulation, and blocking Teff-mediated rejection, leading to tolerance (bottom). The sustained local regulation pictured here could occur via exosomes decorated with interleukin-35 (IL35) subunits (1; Ebi3 and p35), as well as other latent immune-suppressive exosomal products. bi-D, bidirectional; Dnr, donor; Ebi3, Epstein-Barr virus-induced gene 3; HLA-ID, HLA-identical; HSC, hematopoietic stem cell; KTx, kidney transplantation; p35, IL12 subunit p35; Teff, effector T cells; TLI, total lymphoid irradiation; Treg, regulatory T cells.

The permanent establishment of BDIR within the kidney transplant may help account for the extraordinary success of HLA-ID kidney grafts in the Stanford tolerance protocol depicted in the bottom panel of the proposed model (Figure 2). The combination of donor HSC and kidney transplantation with limited recipient HSC ablation^16^ succeeds in inducing long-term, drug-free tolerance in the HLA-ID sibling D-R pair. We would suggest that HLA-ID sibling pairs “work” as subjects for this novel transplantation protocol, not from simply having “only” multiple minor histocompatibility differences from their donor, but also from a preexisting BDIR that is permanently established after concomitant HSC transplantation. In other words, BDIR prevents donor-specific T effector cells (upper left) from accessing the kidney, but eventually T effectors derived from the host bone marrow gain access to the graft, causing rejection. Our proposed model suggests that when combined with donor HSC transplantation and total lymphoid irradiation coverage, recipients of an HLA-ID sibling kidney transplant benefit not only from preexisting,^8^ but also from the permanent establishment of posttransplant, local BDIR.

While some HLA-mismatched LRD pairs have been transplanted successfully under the Stanford protocol, others have rejected their grafts,^6^ consistent with the partial BDIR status of HLA-haploidentical D-R pairs.^7,16^ However, the universal success of the Stanford protocol in HLA-ID sibling D-R pairs (Figure 2, bottom) might be that this procedure, along with the principle of “infectious tolerance,”^17^ perpetuates the lymphoid components of BDIR within the graft. The findings presented here may be relevant to ongoing clinical kidney transplant tolerance trials and tolerogenic immunotherapy in deceased donor organ transplantation.

## ACKNOWLEDGMENTS

The authors acknowledge the contributions of Herman Waldmann (Oxford, United Kingdom) and Colin Anderson (University of Alberta, Edmonton, AB, Canada) for helpful discussions of the data, as well as Sue Medaris, UW Medical Illustration.

## References

[1] Sullivan JA Tomita Y Jankowska-Gan E. Treg-cell-derived IL-35-coated extracellular vesicles promote infectious tolerance. Cell Rep. 2020;30:1039–1051.e5. 31995748 10.1016/j.celrep.2019.12.081 PMC7042971

[2] Bansal S Sharma M R R. The role of exosomes in allograft immunity. Cell Immunol. 2018;331:85–92. 29907298 10.1016/j.cellimm.2018.06.003 PMC6092208

[3] Zeng F Chen Z Chen R. Graft-derived extracellular vesicles transported across subcapsular sinus macrophages elicit B cell alloimmunity after transplantation. Sci Transl Med. 2021;13. 10.1126/scitranslmed.abb0122 PMC8939235 33731430

[4] Chen R Powell JS Shufesky WJ. Transplants foster B cell alloimmunity by relaying extracellular vesicles to follicular dendritic cells. Cell Rep. 2025;44:115832. 40516053 10.1016/j.celrep.2025.115832 PMC12281710

[5] Smyth LA Ratnasothy K Tsang JYS. CD73 expression on extracellular vesicles derived from CD4+ CD25+ Foxp3+ T cells contributes to their regulatory function. Eur J Immunol. 2013;43:2430–2440. 23749427 10.1002/eji.201242909

[6] Burlingham WJ Jankowska-Gan E Fechner JH. Extracellular vesicle-associated GARP/TGFbeta:LAP mediates “infectious” allo-tolerance. Transplant Direct. 2023;9:e1475. 37250483 10.1097/TXD.0000000000001475 PMC10212611

[7] Jankowska-Gan E Sheka A Sollinger HW. Pretransplant immune regulation predicts allograft outcome: bidirectional regulation correlates with excellent renal transplant function in living-related donor-recipient pairs. Transplantation. 2012;93:283–290. 22186938 10.1097/TP.0b013e31823e46a0 PMC3366360

[8] Claas FH Gijbels Y van Der Velden-de Munck J. Induction of B cell unresponsiveness to noninherited maternal HLA antigens during fetal life. Science. 1988;241:1815–1817. 3051377 10.1126/science.3051377

[9] Cai J Lee J Jankowska-Gan E. Minor H antigen HA-1-specific regulator and effector CD8+ T cells, and HA-1 microchimerism, in allograft tolerance. J Exp Med. 2004;199:1017–1023. 15067036 10.1084/jem.20031012 PMC2211880

[10] Hildenbrand K Bohnacker S Menon PR. Human interleukin-12alpha and EBI3 are cytokines with anti-inflammatory functions. Sci Adv. 2023;9:eadg6874. 37878703 10.1126/sciadv.adg6874 PMC10599630

[11] Tripathi S Martin-Moreno PL Kavalam G. Adenosinergic pathway and linked suppression: two critical suppressive mechanisms of human donor antigen specific regulatory T cell lines expanded post transplant. Front Immunol. 2022;13:849939. 35371066 10.3389/fimmu.2022.849939 PMC8968184

[12] Schneider E Winzer R Rissiek A. CD73-mediated adenosine production by CD8 T cell-derived extracellular vesicles constitutes an intrinsic mechanism of immune suppression. Nat Commun. 2021;12:5911. 34625545 10.1038/s41467-021-26134-w PMC8501027

[13] Choi JK Mbanefo EC Yadav MK. Interleukin 35-producing B cells prolong the survival of GVHD mice by secreting exosomes with membrane-bound IL-35 and upregulating PD-1/LAG-3 checkpoint proteins. Theranostics. 2025;15:3610–3626. 40093899 10.7150/thno.105069 PMC11905137

[14] Kang M Yadav MK Mbanefo EC. IL-27-containing exosomes secreted by innate B-1a cells suppress and ameliorate uveitis. Front Immunol. 2023;14:1071162. 37334383 10.3389/fimmu.2023.1071162 PMC10272713

[15] Rojas C Campos-Mora M Cárcamo I. T regulatory cells-derived extracellular vesicles and their contribution to the generation of immune tolerance. J Leukoc Biol. 2020;108:813–824. 32531824 10.1002/JLB.3MR0420-533RR

[16] Scandling JD Busque S Dejbakhsh-Jones S. Tolerance and withdrawal of immunosuppressive drugs in patients given kidney and hematopoietic cell transplants. Am J Transplant. 2012;12:1133–1145. 22405058 10.1111/j.1600-6143.2012.03992.x PMC3338901

[17] Qin S Cobbold SP Pope H. ‘Infectious’ transplantation tolerance. Science. 1993;259:974–977. 8094901 10.1126/science.8094901

